# Improving Early Diagnosis of Child Neglect for a Better Response in Healthcare Settings

**DOI:** 10.3390/children8100859

**Published:** 2021-09-27

**Authors:** Silvia Herrero-Roldán, Inmaculada León, Juan Andrés Hernández-Cabrera, María José Rodrigo

**Affiliations:** 1 Instituto Universitario de Neurociencia, Universidad de La Laguna, 38200 La Laguna, Spain; roldansh@gmail.com (S.H.-R.); jhernand@ull.edu.es (J.A.H.-C.); mjrodri@ull.es (M.J.R.); 2 Facultad de Psicología, Universidad de La Laguna, 38200 La Laguna, Spain

**Keywords:** child neglect, life adversity, mental health, mother–child resilience, prevention, intervention

## Abstract

Early diagnosis of child neglect is an ongoing challenge with consequences of the child’s safety, health, and effective referral for intervention. This study aims to obtain a selected set of family, maternal, and dyadic variables of the immediate caregiving environment for diagnosis, preventive, and intervention responses in healthcare settings. Mothers and their children under five years old: 51 in the neglected group (NG) and 89 in the non-neglected control group (CG), were recruited through pediatric primary care services and social services in Spain. Family demographics, adverse events, childhood maltreatment, maternal psychopathologies, personality variables, and observed mother–child interactions were assessed. Gradient boosting analyses were applied for the contributor’s relative importance (*RI*), followed by logistic regression and discriminant analyses for those with higher *RI*. Parametric analyses showed high diagnostic accuracy (80–82% of NG and 92% of CG) for risky factors of child neglect: having a physically neglected and depressed mother, living in families in need of financial assistance, and large families; and for protective factors: having an older mother and showing higher mother–child emotional availability. Identifying a select group of features makes early diagnosis and preventive intervention more effective for mitigating the impact of child neglect and building mother–child resilience.

## 1. Introduction

Research has demonstrated the significant short- and long-term impact of adverse childhood experiences, such as abuse and neglect, and the social determinants, such as the family socioeconomic disadvantage, on healthy child development and wellbeing. [1,2]. Among the adverse childhood experiences, neglectful caregiving is the most common and severe form of child maltreatment (approximately 75% of maltreated children are neglected), consisting of the caregivers’ failure to provide the child with food, clothing, shelter, medical care, supervision, or emotional support [3,4,5]. In Spain, child neglect represents 52% of all notifications of suspected child maltreatment, based on data from the Unified Registry of Cases of Suspected Child Abuse and Neglect [6]. Being severely neglected in the early years of life disrupts the establishing of a child’s secure attachment and carries a cumulative risk during adolescence and adulthood for physical and mental health, behavioral problems, and neurobiological alterations [7,8,9].

Primary health services, especially pediatric services, are considered a universally accessed and non-stigmatized setting. That makes them particularly suited for being engaged in a possible case of child neglect in two main situations. The first is to provide ongoing medical care and guidance for neglected children with child protection services (CPS) records [10]. The second one is to identify a child neglect case early in the absence of CPS records. However, screening young children for risk factors, such as an adverse childhood experiences and protective factors, does not occur regularly [11]. In the absence of a proper screening, primary care providers often struggle with deciding when an action (or lack of action) by a caregiver constitutes inadequate childcare and is neglectful [12]. Having well-founded knowledge about risk factors and relevant absent protective factors would increase the likelihood of detecting a proper case and not a false positive. The evidence-based grounds would give more guarantees for starting an alert protocol that activates the social services and/or CPS to substantiate the case. Therefore, achieving an early diagnosis of child neglect can positively affect the child’s safety, health, and wellbeing and subsequent effective referral for additional intensive intervention by other services.

According to the child protection system (CPS), the diagnosis of child neglect is guided by a set of indicators of the disregard of the child’s needs [13] and based on a safety assessment of the imminent risk of harm to the child [14]. However, a diagnosis solely based on child indicators and protection from imminent risk does not consider the multidimensional nature of child neglect comprising multiple variables associated with distal and proximal features of the caregiving environment [3,15]. This study aims to obtain a select group of variables of the proximal/immediate caregiving environment, among those previously identified as related to child neglect, to make an early diagnosis and preventive intervention more feasible and effective for the professionals.

There is ample evidence that child neglect is associated with family and life adversity, parental, and dyadic factors operating within the immediate caregiving environment. Multiple stressors within the family environment (e.g., poverty, social isolation), and the own mothers’ history of childhood maltreatment and negative life events [3], are related to child neglect. In turn, parents’ mental/psychiatric problems (e.g., depressive symptoms) are also among the most relevant risk factors of child neglect [16]. Less evidence has been obtained about other factors, such as maternal personality traits and adult attachment orientation. Alexithymia refers to difficulty in recognizing and describing one’s emotions, differentiating mental states from bodily sensations, and minimizing emotional experience by focusing attention externally [17], and is higher in mothers with neglectful caregiving [18]. Empathy refers to the appropriate perception, understanding, and experience of an infant’s emotional states [19], and is one of the abilities for providing caring responses to an infant’s needs, which is lower in mothers with neglectful caregiving [20,21]. Social anhedonia refers to deficits in the ability to experience pleasure from social (child or adult) stimuli and poor social engagement [22], and is higher in mothers with neglectful caregiving [23]. In turn, mothers’ adult attachment style may also be related to child neglect, since insecure attachment orientation has been related to child maltreatment perpetration/child abuse potential [24].

In addition to the family and parental factors, dyadic factors concerning responsive mother–child interaction may also be associated with early child neglect by disrupting the infant-mother attachment process. Evidence shows that the quality of this early sensitive interaction in daily exchanges is crucially associated with infant neglect [21,25] and also predicts the infant’s attachment quality [26]. From a broader view, early relational health is a multidimensional concept that emphasizes the importance of earliest relational experiences and caregiver-child interactions that build lifelong health, early learning, social-emotional capacities, self-regulation and resilience [27]. Therefore, lower sensitive-responsive interactions in the mother–child neglectful dyads can be taken as an early signal of disruption in functional caregiving that involves a child’s lower relational health. In particular, the dyadic emotional availability (EA) during mother–child exchanges in a free play task [28,29], may be understood as a subtle and intimate marker of neglectful parenting.

Despite the previous evidence, the crucial joint contribution of family and life adversity, parental, and dyadic variables to the early diagnosis of child neglect, leading to more effective intervention strategies is less explored. To fill this gap, the objective of this study is to examine the respective contributions of family demographics (e.g., mother’s age, financial assistance, number of children, single-parent family) and life adversity (mother’s childhood maltreatment and negative life events), mother’s psychopathological conditions (e.g., depression, anxiety), personality traits (alexithymia, empathy, and social anhedonia), and adult attachment styles, as well as observed mother–child emotional availability, to the differential diagnosis of the neglected group (NG) versus the non-neglected control group (CG) of children, according to pediatricians and CPS’s identification. We used a two-step methodological procedure to provide data-driven and parametric validation to guarantee the rigorous selection of a group of variables limited in size but sufficient to support accurate early diagnosis of child neglect and give insights for intervention targets.

## 2. Materials and Methods

### 2.1. Participants

One hundred forty mothers (51 in the neglected child group (NG) and 89 in the non-neglected child control group (CG)) and their respective children were recruited through the same municipal social services and primary health centers in Tenerife, Spain. Specific inclusion criteria for the child in the NG were: being under five years old, having been registered in the last 12 months as a pure and substantiated case of neglect by child protective services (CPS) according to the reports of the social services, and complying with all the indicators of the Maltreatment Classification System (MCS) for severe neglect [13] according to the pediatrician of the primary health center. Thus, a child in the NG should score positively on physical neglect (inadequate food, hygiene, clothing, and medical care), lack of supervision (child is left alone or in the care of an unreliable caregiver), and educational neglect (lack of cognitive and socio-emotional stimulation and lack of attention to the child’s education). The child’s inclusion criteria in the CG were: having negative scores in the same indicators of the MCS and absence of CPS records. None of the neglected or control infants had been placed in foster care at any point in their history, nor had they been born prematurely or suffered perinatal or postnatal medical complications. Although the size of the two groups is moderate, previous studies working with smaller sample sizes (25–30 mothers) per group have obtained significant differences in many of our study’s variables [21,25]. Besides increasing the number of mothers in the neglect group, we double the number of control mothers to obtain the highest heterogeneity in the normative population, including that source of variance in the estimated models.

Table 1 (upper part) shows the sociodemographic profile and statistical differences between both groups. Mothers in the NG were younger and with a higher number of pregnancies than mothers in the CG, and the target child had a similar mean age in both groups. Moreover, NG mothers were less likely than mothers in the CG to live in two-parent families and more likely to show a lower educational level and to receive financial assistance than those in the CG.

### 2.2. Instruments

#### 2.2.1. Family and Life Adversity

We collected socio-demographic data on maternal age, age of the target child, number of children, mother’s educational level, family structure, financial assistance from institutions, employment status, and family’s place of residence.

The own mothers’ history of childhood abuse or neglect in the family was assessed using the Childhood Trauma Questionnaire—Short Form [30,31], which contains 28 items rated on a 5-point Likert scale, in five subscales: physical neglect (α = 0.71), emotional abuse (α= 0.92), physical abuse (α = 0.88), sexual abuse (α = 0.94), and emotional neglect (α = 0.93). The total score of each subscale was obtained by adding the score of the corresponding items.

The Life Stress Scale (LSS) was used to assess the mothers’ adverse life events experienced in the family, making an adaptation of adverse childhood experiences to our risk population [32]. It comprises 16 negative events related to this population (e.g., divorce, economic pressure, chronic illness, eviction, unwanted pregnancy) rated (no/yes occurrence) and its emotional impact on the participant on a 3-point Likert scale. The total score was obtained by adding the emotional impact of the events that the mother had suffered (α = 0.77).

#### 2.2.2. Maternal Individual Features

The mothers’ psychopathological conditions were assessed using the Mini-International Neuropsychiatric Interview [33]. It assesses on a categorical scale (no/yes) symptoms of the 16 most common psychiatric disorders in DSM-IV and ICD-10: major depressive episode, dysthymia, hypo/manic episode, suicidality, general panic disorder, agoraphobia, social phobia, obsessive-compulsive disorder, post-traumatic stress disorder, alcohol dependence/abuse, drug dependence/abuse, psychotic disorders, anorexia nervosa, bulimia nervosa, generalized anxiety disorder and antisocial personality. Scores obtained for each disorder correspond to a cumulative scoring of symptoms. An additional (Yes/No) question answered by the social workers about the mothers’ current alcohol and drug use, was included in this set of variables.

Alexithymia was assessed using the Toronto Alexithymia Scale [34], which contains 20 items rated on a 6-point Likert scale, in three subscales: TAS—F1, difficulty describing feelings to others (α = 0.73); TAS—F2, difficulty identifying feelings and distinguishing them from bodily sensations of emotion (α = 0.90); and TAS—F3, externally oriented thinking to focus on the simpler and external aspects of the events rather than the psychological correlates (α = 0.50).

Empathy was assessed using the Interpersonal Reactivity Index [19,35], which contains 28 items rated on a 5-point Likert scale in four subscales: perspective-taking to adopt the psychological point of view of others (α = 0.77); fantasy to identify with fictional characters (α = 0.69); empathic concern to others’ emotions with feelings of warmth and concern for others (α = 0.59); and personal distress with feelings of anxiety and discomfort in social settings (α = 0.77).

Social anhedonia was assessed using the Revised Social Anhedonia Scale [22,36], which contains 40 items in a categorical (true/false) scale to assess deficits in the ability to experience pleasure from other social stimuli, such as talking and exchanging expressions of feelings (α = 0.81).

The adult attachment was assessed using the Attachment Style Questionnaire [37,38], which contains 40 items rated on a 6-point Likert scale in five subscales: ASQ—F1, confidence or security of attachment (α = 0.72); ASQ—F2, need for approval and acceptance (α = 0.73); ASQ—F3, preoccupation with relationships about whether partners’ feelings of love are deep and lasting (α = 0.71); ASQ—F4, discomfort with high levels of intimacy and dependence on partners (α = 0.62); and ASQ—F5, relationships as secondary to protect themselves, by emphasizing achievement and independence (α = 0.72) for our sample. For the personality and the adult attachment assessment, each subscale’s total score was obtained, adding the corresponding items’ score.

#### 2.2.3. Dyadic Mother–Child Interaction

The mother–child emotional availability was assessed using the Infancy to Early Childhood Version Scale [29]. Emotional availability is defined as the mother and child ability to read and respond appropriately to each other’s communications during a play task. The dyadic performance is predictive of the mother’s reported child attachment [39]. Two external observers, blind to the mothers’ grouping, made the videos’ ratings and the inter-rater reliability were calculated. For the mother’s behavior: *sensitivity* (the mother shows contingent responsiveness to child signals and demands, (Kappa score (*K*) = 0.94); *structuring* (the mother appropriately facilitates the child’s play, *K* = 0.90); *non-intrusiveness* (the mother can support the child’s play without being over directive and/or interfering, *K* = 0.87); *non-hostility* (the mother can behave with the child in a way that is not rejecting or antagonistic, *K* = 0.92). For the child’s behavior: *responsiveness* (the child’s ability and interest in exploring on his or her own and in responding to the parent’s bids, *K* = 0.92), and *involvement* (the child’s ability and willingness to engage the mother in interaction, *K* = 0.86). A principal component analysis yielded a single factor structure: *KMO* = 0.84, Eigenvalue = 4.49, with an explained variance of 75%. The coefficient score in this factor was used as a measure of dyadic emotional availability (EA).

For the sake of brevity, Table 1 (bottom) shows the study variables showing significant differences between the control and neglect groups.

### 2.3. Procedure

Pediatricians attending the families were contacted to obtain their evaluation of the indicators of the Maltreatment Classification System. Social workers reported on the participants’ family characteristics and asked mothers to contact them by phone. The mothers contacted by our collaborator were informed about the study (“to know more about mother–child relationships”) and the procedure. Next, the mothers were visited at their homes, first collecting their responses to the questionnaires, and then giving a gift to the child to be used for a video recording of the mother–child play interaction. The mothers were instructed to use the toy and play with the child as they usually do. At the end of the session, mothers were given monetary compensation (EUR 30).

### 2.4. Ethical Considerations

The Ethics Committee of the University of La Laguna approved this study. Each participant received written and oral information regarding the study. The mothers signed two informed consents both for their participation and that of their children. Following data protection laws, to guarantee confidentiality, we create a dataset with identifying information and a code assigned to each participant, and another one without identifying information and with the code and data of the different variables of interest.

### 2.5. Plan of Data Analysis

Data were analyzed using R [40]. Missing data, representing 5% or less of the total data collected, were estimated through random forest missing value estimation with the whole set of variables as predictors [41]. We used gradient boosting (GB) analyses to obtain the data-driven evidence, a non-parametric and machine learning approach [42,43]. This technique helps to investigate the multivariate association and reduce the dimensionality within each of the four sets of variables: demographic characteristics, maternal life adversity, psychopathological conditions, and personality traits plus adult attachment style, according to their relative importance to the NG versus CG profiles. The whole sample was randomly divided into a training sample, used to estimate the model that best classifies the records, and an independent sample made up of the remaining records, called “out of bag” (*OOB*), on which the goodness of the estimated model (sensitivity and specificity of classification) is tested. Each predictor’s contribution is informed as *RI* (relative importance), and the larger the *RI*, the greater the contribution, indexing the relative error in the classification when this predictor is excluded from the estimated model. The *RI* values do not have an upper limit; thus, they must be interpreted considering the *RIs* of the other variables included in the model.

The variables selected from the four GB analyses by their relative importance (*RI)* were submitted to two parametric analyses. The dyadic emotional availability score was also added. The first parametric model, a multiplicative stepwise logistic regression, was used to identify the group of variables that best modelled the risk of being neglected versus a non-neglected child. The second parametric model, an additive stepwise linear discriminant analysis, was used to obtain the best variable combination to classify the children (NG and CG). The diagnostic accuracy for both parametric approaches was quantified by the contributors’ predictive and discriminant values and by the standard measures of sensitivity (the potential to identify NG condition correctly) and specificity (the ability to identify CG condition correctly).

## 3. Results

### 3.1. Selecting and Ruling out Variables

After applying gradient boosting procedures (GB) on each of the four groups of variables, one set of each (four) was selected based on their highest *RI* values to ensure each group’s equivalent representation in the final confirmatory analyses. As shown in Table 2 (left), the first GB, estimated on the demographic variables, showed an important sensitivity of 83.3% to detect the NG, and a relatively lower specificity (68.8%) to detect the CG. According to the relative importance values (*RI*), age of the mother, number of children, institutional financial help needed, and family structure (one- or two-parent family) were the most important variables to distinguish the two groups.

**Figure 1:**
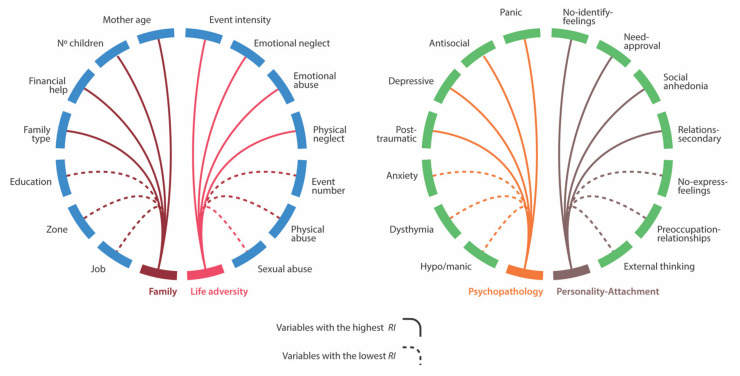
Set of variables resulting from the gradient boosting analyses of family and life adversity variables (left), and of maternal psychopathology and personality-attachment variables (right) that distinguish between the neglected group and the non-neglected control group. The solid/dotted connection lines represent variables that were or were not selected for follow-up confirmatory analyses based on their relative importance values (RI).

The second GB, estimated on the family life adversity variables (negative life events and types of own childhood maltreatment), showed similar sensitivity and specificity to detect the NG and the CG (75% and 72% respectively) in Table 2 (right). The most important variables to distinguish between the two groups were the mothers’ emotional impact of negative events and the history of their own emotional neglect, emotional abuse, and physical neglect. Figure 1 (left) depicted the results of the first and the second gradient boosting analyses.

The third GB, estimated on the set of mothers’ psychopathological conditions in Table 3 (left), showed a relatively lower sensitivity of 72% to detect the NG and a higher specificity of 83.3% to detect the CG. General panic disorder, antisocial personality, major depressive episode, and post-traumatic stress disorder were the most important variables (*RI*) to distinguish the two groups. Although substance abuse is a variable that clearly characterizes mothers with negligent behavior, as the univariate analysis shows (19% of mothers in the neglect group, it has not maintained its statistical relevance when competing with other psychopathological variables, which absorbed all the explanatory power.

The fourth GB, estimated on the set of personality and attachment variables in Table 3 (right), showed good sensitivity of 75% to detect the NG and lower specificity of 61.5% to detect the CG. The variables with the highest relative importance were alexithymia TAS—difficulty identifying feelings, adult attachment ASQ—need for approval, social anhedonia, and adult attachment ASQ—relationships as secondary. Psychopathological variables with *RI* value of zero, and IRI variables and ASQ variables (confidence and discomfort with closeness) with *RI* lower than 6.82, were not shown. Figure 1 (right) depicted the results of the third and the fourth gradient boosting analyses.

### 3.2. Modeling the Risk of Child Neglect

The variables resulting from the previous analyses, including the factor score of emotional availability in each group, were submitted to logistic regression to obtain parametric evidence of their role in modelling the risk of child neglect. Table 4 (left) shows the exponential *b* parameters (*Exp(b)*) estimated in the logistic regression for the variables that were shown to be significant in the stepwise procedure implemented. The logistic model’s sensitivity to classify the neglected group was 80.3%, and the specificity to classify the non-neglected group was 91.9%.

The emotional availability factor (with an *Exp(b)* < 1) showed an important protection effect, dividing the risk of child neglect by a factor of 3.57 (1/0.28). The major depressive episode (with an *Exp(b)* >1) increased the risk of child neglect by a multiplicative factor of 1.3, 30% by a unit of increment. The family’s financial help multiplied by a factor of 7.08 the risk of child neglect. The maternal childhood physical neglect (with an *Exp(b)* >1) increased the risk of child neglect by a multiplicative factor of 1.67, 67% by a unit of increment. The mothers’ age had an important protection effect, dividing the risk of child neglect by a factor of 1.21 (1/0.82) where the older the mother, the lower the risk of child neglect is. Finally, the number of children had a high impact, since each increment unit multiplies by three, the risk of being a neglected child.

### 3.3. Validating the Diagnostic Accuracy of the Selected Variables

Discriminant analysis validated the diagnostic accuracy of the previous parametric analysis yielding a statistically significant function, *Wilks’ Lambda* = 0.44, *F*(1, 136) = 168.44, *p* < 0.001, *η*^2^ = 0.34 (Table 4 right). The structure coefficient (correlation between each variable and the discriminant function) and the standardized coefficient (relative importance of each variable in predicting group assignment from the function) go in the same direction as the logistic regression outcomes.

## 4. Discussion

This study allows for a more accurate, early preventive diagnosis of child neglect resulting from the combination of family and life adversity, maternal, and dyadic factors, featuring the immediate caregiving environment using two sources of evidence. The selection of risk and protective factors showed high classificatory proficiency, correctly classifying the neglected group over 80% and the control group over 92% of the time (regression model), and the neglected group over 82% and the control group over 92% of the time (discriminant model). The child’s risk of being neglected increases when the family faces substantial economic hardship and has many children, as shown in previous studies [3,44]. The risk also increases when the mother has experienced childhood physical neglect, supporting the cycle of intergenerational transmission of neglect [45]. Moreover, this finding points to the specificity of suffering physical neglect as the link connecting maternal history and maternal neglect, more than through the experience of emotional neglect and abuse. Maternal depression symptoms also emerge as a risk factor, since it increases the self-focus and psychological distancing that disrupt the caregiving role [16]. Although substance abuse is a variable that clearly characterizes mothers with negligent behavior, it has not maintained its statistical relevance when competing with other psychopathological variables, which absorbed all the explanatory power.

As a novelty, our findings reveal the existence of protective factors. The child’s risk decreases when the child is born later in the mother’s life, and when there is higher mother–child emotional availability (EA). Younger mothers are more likely to have children referred to child protective services for abuse and neglect [46]. Importantly, lower capacities in EA have shown brain correlates in mothers with neglectful caregiving consisting of volume reductions in the white matter tract (inferior longitudinal fasciculus), which is involved in the processing of emotional faces [25], as well as volumetric differences in gray and white matter in empathy-related regions [21]. These brain differences in critical areas for adequate parenting support the importance of emotional availability as a relevant indicator of a lower relational health in the early diagnosis of child neglect.

Data-driven evidence also revealed the importance of other variables, offering a coherent, complementary view of maternal contributors of child neglect. Besides depression symptoms, vulnerability to general panic disorder and post-traumatic stress disorder and the presence of antisocial personality disorder were also critical diagnostic variables. These findings support the internalizing and externalizing patterns linked to early social stress [47] and maternal childhood maltreatment [48], both features that characterize the profile of mothers with neglectful behavior. Results from personality variables (alexithymia and social anhedonia) and adult insecure attachment also suggest that mothers with neglectful caregiving present maladaptive dispositional tendencies associated with perceived own emotional distress and with unstable intimate and social relationships with the child and other adults [21,23]. The mother’s pre-existing insecurity attachments is a risk factor for intergenerational transmission of violence, trauma, and relational disturbances [49]. In sum, mothers with neglectful behaviors carry a heavy mental burden linked to childhood maltreatment, psychopathologies, and maladaptive dispositions, disturbing the necessary attentional focus on the child’s care.

Although this study assessed a broad set of variables, contrasting their diagnostic relevance against an externally validated categorization of severe child neglect, limitations should be mentioned. First, distal variables in the macrosystem (e.g., social support, neighborhood characteristics), father-related variables and child characteristics (e.g., temperament) were not tested at the expense of wider coverage maternal-related variables. Second, the self-reported questionnaires could be subject to recall bias. Third, the cross-sectional design does not allow for assessing causal relations among contributors and child neglect. Finally, the neglect cases and comparisons in this study were predominantly from mothers in lower socioeconomic strata, as is usually the case in public healthcare and social services. These findings may not be generalizable to cases of neglect from middle- or upper-class families.

## 5. Conclusions

This study brings novelty to the timely issue of the early detection of child neglect by offering a limited set of risk and protective factors with a high diagnostic value that have been rigorously selected and are supported by multivariate modelling. Our findings can help evidence-based early identification, prevention, and intervention strategies to mitigate the negative impact of child neglect and build mother–child resiliency. First, screening young children in primary care for child neglect must emphasize at least the maternal history of physical neglect, maternal depressive symptoms, and stressful family circumstances affecting the caregiving scenario, in addition to child-related factors. This screening can ensure the child’s proper development and is essential to break the cycle of intergenerational transmission of child neglect.

Second, the protective role of dyadic emotional availability should also be screened paying attention to the quality of early mother–child interaction as a basis for developing mother–child resiliency. Therefore, a specific mother–child intervention for mothers who have experienced trauma in their life and have inappropriate representations of their child’s basic needs would be helpful and preventive for the child’s social-emotional development. This intervention also alleviates maternal distress and increases self-regulatory abilities (e.g., self-confidence and self-efficacy) in managing their caregiver role [49]. In this regard, there is evidence that video training interventions, exposing mothers to their interactions with the child, improve their sensitivity to the child’s emotional expressions of their needs and results in healthier infant attachment [50]. Family-focused preventive actions in pediatric primary care using evidence-based programs have also shown parental improvements in health promotion activities with the child, parental self-regulation in their caregiver role, and satisfaction with the service [51]. These positive changes were obtained after parents participated in “Gaining health and wellbeing from birth to three”, a web-based hybrid program including group workshops and individual supports delivered by healthcare practitioners. All of these are interventions that help improve the infant’s relational health as a protective factor against poorer developmental outcomes [27].

Finally, although identifying child neglect falls well within a broad pediatric diagnosis to protect children and enhance their health and wellbeing, the proper fulfilment of this task requires a coordinated and systems-based approach [11]. This approach improves both intra-agency referral practices (with the gynecologist, the family doctor, the obstetric and mental health professionals) and inter-agency referral practices (with the protection system, childhood education, and social and community services). Both engagements will increase the universal reach of preventive strategies and the opportunity for a broader and more intensive intervention. The perspective adopted in this study offers a promising venue to delineate better the factors that contribute to the early preventive diagnosis of child neglect and to develop new directions for more effective targeted interventions.

## Figures and Tables

**Table 1:** Demographic characteristics, and study variables showing differences between the control and neglect group.

	Control Group (*n* = 89) *M* (*SD*) or %	Neglect Group (*n* = 51) *M* (*SD*) or %	*t* (138)/*χ2*
**Family characteristics**			
Age of mother	34.04 (6.09)	30.67 (7.38)	2.92 **
Number of children	1.67 (0.75)	2.49 (1.28)	−4.15 ***
Mean age of the target child	3.05 (1.55)	2.58 (1.59)	1.69
One-or two-parent family (Two-parent family %)	73	49	7.13 **
Educational level (%):			18.75 ***
Primary	43	80	
Secondary school	51	18	
>Secondary school	7	2	
Urban/rural area (Rural areas %)	27	43	3.15
Job stability (Unemployment %)	58	71	1.57
Institutional financial help %	26	68	21.81 ***
**Life adversity**			
Intensity of negative events	11.55 (7.66)	16.76 (8.65)	−3.7 ***
Childhood emotional neglect	8.83 (4.24)	10.98 (5.73)	−2.34 *
Childhood emotional abuse	7.25 (4)	11.39 (6.70)	−4.03 ***
Childhood physical neglect	5.83 (1.7)	8.27 (4)	−4.15 ***
Number of negative events	5.09 (3.13)	7.1 (3.35)	−3.56 ***
Childhood physical abuse	6.13 (2.23)	8.33 (4.81)	−3.08 **
Childhood sexual abuse	5.89 (2.96)	8.98 (5.95)	−3.47 ***
**Psychopathology**			
General panic disorder	2.33 (4.89)	5.74 (6.84)	−3.14 **
Antisocial personality	0.46 (1.17)	1.80 (2.56)	−3.53 ***
Mayor depressive episode	0.73 (2.08)	3.69 (3.95)	−4.96 ***
Post-traumatic stress disorder	0.78 (2.29)	2.40 (4.25)	−2.45 *
Generalized anxiety disorder	0.76 (1.93)	2.35 (3.51)	−2.99 **
Hypo/manic episode	0.56 (1.91)	1.85 (3.35)	−2.51 *
Substance abuse %	2	19	9.51 **
**Personality-Attachment**			
TAS—Difficulty identifying feelings	15.6 (7.48)	19.90 (9.93)	−2.69 **
ASQ—Need approval	17.86 (5.10)	20.19 (6.48)	−2.35 *
Social Anhedonia	8.31 (4.41)	11.47 (5.76)	−3.39 **
ASQ—Relation as secondary	13.11 (4.03)	15.34 (5.21)	−2.83 **
TAS—Difficulty expressing feelings	14.03 (5.64)	16.29 (6.08)	−2.22 *
ASQ— Preoccupation with relationships	22.09 (5.81)	25.12 (7.12)	−2.59 *
**Emotional availability**			
EA factor	0.64 (0.55)	−0.64 (0.93)	−5.76 ***

Note: *M*: mean score, *SD*: standard deviation; * *p* ≤ 0.05; ** *p* ≤ 0.01; *** *p* ≤ 0.001.

**Table 2:** Gradient boosting analyses estimated on the two sets of family and life adversity variables showing good “out of bag” (*OOB*) sensitivity and specificity, and the relative importance (RI) of the variables for the distinction of the neglected group (NG) and the non-neglected control group (CG).

Family and Life Adversity Variables					
*GB1: Family demographics*			*GB2: Life adversity*		
**NG-*OOB* (%)**	**CG-*OOB* (%)**		**NG-*OOB* (%)**	**CG-*OOB* (%)**	
Sensitivity: 83.3	Specificity: 68.8	*RI*	Sensitivity: 75	Specificity: 72	*RI*
**Age of mother ^a^**		**43.40**	**Intensity of negative events**		**24.46**
**Number of children**		**15.76**	**Childhood emotional neglect**		**18.97**
**Institutional financial help**		**12.34**	**Childhood emotional abuse**		**15.58**
**One-or two-parent family**		**8.93**	**Childhood physical neglect**		**14.02**
Educational level		8.79	Number of negative events		13.18
Urban/rural area		7.59	Childhood physical abuse		9.40
Job stability		3.15	Childhood sexual abuse		4.35

Note. ^a^ Labels in bold indicate the variables selected for the subsequent confirmatory analyses. GB: gradient boosting. *OOB*: out of bag. *RI*: relative importance.

**Table 3:** Gradient boosting analyses estimated on the two sets of maternal variables showing good “out of bag” (*OOB*) sensitivity and specificity, and the relative importance (RI) of the variables for the distinction of the neglected group (NG) and the non-neglected control group (CG).

Maternal Variables					
*GB3: Psychopathological conditions*			*GB4: Personality & attachment variables*		
**NG-*OOB* (%)**	**CG-*OOB* (%)**		**NG-*OOB* (%)**	**CG-*OOB* (%)**	
Sensitivity: 72	Specificity: 83.3	*RI*	Sensitivity: 75	Specificity: 61.5	*RI*
**General panic disorder ^a^**		**21.34**	**TAS ^b^-Difficulty identifying feelings**		**16.3**
**Antisocial personality**		**19.11**	**ASQ ^c^-Need for approval**		**15.68**
**Major depressive episode**		**18.2**	**Social anhedonia**		**10.81**
**Post-traumatic stress disorder**		**17.87**	**ASQ—Relationships as secondary**		**8.37**
Generalized anxiety disorder		13.82	TAS—Difficulty expressing feelings		7.43
Dysthymia		6.4	ASQ—Preoccupation with relationships		6.91
Hypo/manic episode		2.41	TAS—Externally oriented thinking		6.82

Note. ^a^ Labels in bold indicate the variables selected for the subsequent confirmatory analyses. ^b^ TAS (Toronto Alexithymia Scale); ^c^ ASQ (Attachment Style Questionnaire).

**Table 4:** Estimated exponential b parameters in the logistic regression and discriminant coefficients as well as diagnostic accuracy values for the distinction of the neglected group (NG) and the non-neglected control group (CG).

	Logistic Regression		Discriminant Analysis	
*Variables*	*Exp(b)*	*Z*	*Structure* *Coefficient*	*Standard* *Coefficient*
Dyadic emotional availability	0.28	−3.37 ***	−0.52	−0.49
Mother Major depressive episode	1.30	3.00 **	0.44	0.46
Institutional financial help	7.08	2.97 **	0.44	0.38
Mother Childhood physical neglect	1.67	3.24 ***	0.39	0.32
Age of mother	0.82	−3.37 ***	−0.22	−0.44
Number of children	3.01	2.46 **	0.39	0.36
NG Sensitivity (%)	80.39		82.35	
CG Specificity (%)	91.95		91.94	

** *p* ≤ 0.01; *** *p* ≤ 0.001.

## Data Availability

Data that supports the findings of this study are available on request from the corresponding author.
